# Grain quality evaluation of *japonica* rice effected by cultivars, environment, and their interactions based on appearance and processing characteristics

**DOI:** 10.1002/fsn3.2181

**Published:** 2021-02-24

**Authors:** Yujie Xia, Yuying Sun, Jian Yuan, Changrui Xing

**Affiliations:** ^1^ College of Food Science and Engineering Collaborative Innovation Center for Modern Grain Circulation and Safety Key Laboratory of Grains and Oils Quality Control and Processing Nanjing University of Finance and Economics Nanjing China

**Keywords:** cultivars, environments, *japonica* rice, quality evaluation

## Abstract

Appearance and processing characteristics of 45 *japonica* rice samples, collected from different regions in Jiangsu province, were investigated and evaluated in this study. Specifically, the chalkiness degree had been presented significant differences among different cultivars and regions. The average chalkiness degree varied from 6.81% to 15.34% for different regions and from 1.93% to 28.31% for different cultivars. The minimum head rice rate of cultivars from four regions, NJ9108 (HA), was 80.5%. The AC of CNG10, HD5, and PJ surpassed 13.68% and lower than 11.33% for the others. The protein content ranged from 6.1% to 11%, and the taste value was significantly different among cultivars. In addition, the RVA curves of the samples were similar, but the peak viscosities of NG8 and NJ5055 were higher than others, and there were significant differences in RVA traits among regions. Cultivars were the main reasons for the difference in appearance and processing quality of *japonica* rice, while environmental factors had leaded to the change of rice composition, texture, and gelatinization.

## Graphical abstract

We collected 45 high‐quality *japonica* rice samples for quality analysis. Cultivars were the main reasons for the difference in appearance and processing quality of *japonica* rice, and environmental factors had leaded to the change of rice AC content, texture, and gelatinization.

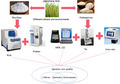

## 1 INTRODUCTION

Rice (*Oryza sativa*) is one of the world's major crops, and more than 1/2 of people eat rice as a staple food. China is the largest rice producer and consumer in the world (Duan & Zhou, 2013; Yan et al., 2015). According to the current trend of rice market development, the eating quality of *japonica* rice and their potential economic value are better than *indica* rice for its better palatability and flavor (Balindong et al., 2018; Nádvorníková et al., 2018; Zeng et al., 2019). It is common to evaluate the grain quality of rice by comprehensive analysis of rice traits (Zeng et al., 2019). Many common indexes including processing appearance, nutritional qualities, cooking, and eating properties are high‐related with rice traits and used in rice quality evaluation (Concepcion et al., 2018; Liu et al., 2013).

With the improvement of people's living standards in recent years, the huge demand for high‐quality rice have shift market attention from quantity advantage to quality advantage (Cheng et al., 2019; Su et al., 2019). However, rice cultivars were bred and planted in specified region, harvesting with different quality (Liu et al., 2015; Takai et al., 2019). Meanwhile, Jiangsu province have obvious environment difference from north to south and this difference effects grain rice quality. At present, the influence and difference among environment and cultivars on grain rice quality have not detailed investigated (Wang, et al., 2015) grain quality, and re). These above phenomena had resulted in superior‐quality rice and ordinary rice coexisted in the market (Liu et al., 2015). Therefore, it is great necessity and worth to enrich rice quality database to analyze the *japonica* rice quality more systematic and comprehensive and understand the relation between environment and cultivars effect on *japonica* rice quality from appearance and processing characteristics. These parameters related to the rice quality are shown in Table 1 and researched thoroughly.

**TABLE 1:** Rice quality parameters

Rice quality	Details	Reference
Appearance quality	chalkiness degree, head rice rate, yellow‐colored rice, chalky rice rate, grain width, grain thickness, grain length‐to‐width ratio, etc.	Peng et al. (2014) Cheng et al. (2019)
Composition	AC, protein content, fat content, vitamin, etc.	Balindong et al. (2018) Fitzgerald et al. (2009)
Texture analyzer	hardness, resilience, chewiness cohesiveness, gumminess, etc.	Billiris et al. (2012)
RVA	gelatinization temperature, peak viscosity, trough viscosity, Final viscosity, etc.	Cozzolino (2016)

The objectives of this study were to (a) compare the appearance quality and processing quality of *japonica* rice samples produced in RuDong, Hai’An, XingHua, and LiShui; (b) analyze the quality differences among regions and cultivars; (c) explore the influence of environment and cultivars on rice quality; and (d) provide a valuable analysis and reference for the establishment of *japonica* rice quality database and rice quality evaluation.

## 2 MATERIALS AND METHODS

### 2.1 Sample collection

A total of 45 autumn harvest *japonica* rice samples were collected from four regions in Jiangsu province (RuDong, Hai’An, XingHua, and LiShui) in 2019. The samples were divided into 11 groups according to their cultivars (Table S1). All samples were collected on‐site to ensure the pureness and traceability (Table S2). Then, the moisture content of all samples was dried to <15.5%. The samples were protected from light for 30 days at room temperature before experiments.

### 2.2 Experimental method

The chalkiness degree (CD), head rice rate (HRR), protein content, AC (AC), taste value, texture, and RVA of the samples were measured. Relevant information of accumulated temperature, rainfall, and relative humidity was obtained from Hefeng weather (https://www.heweather.com/).

#### 2.2.1 Determination the appearance quality and rice taste value

Paddy huller (BLH‐3250B, Grain Instrument Factory) was used to grind 200 g rice for three times to get brown rice. Subsequently, the obtained brown rice was shelled by rice polisher (LTJM‐6688, Grain Instrument Factory) twice (each time in 90 s) to get the milled and polished rice. The polished rice was grounded by a grinder (FW100, TaiSiTe) for the 30 s to get rice flour.

CD and HRR were measured by the Rice Appearance Quality Tester (JMCT 12, Dongfu Jiuheng). Each group of samples was measured three times in parallel. The taste value was measured by a taste meter (JSWL, Dongfu Jiuheng). The taste meter was based on the combination of near‐infrared spectrum (NIR) principle and related computer application software.

#### 2.2.2 Component determination

The protein content was tested following the procedures: 6.5 g K_2_SO_4_ (AR), 0.5 g CuSO_4_ (AR), and 12 ml H₂SO₄ (AR) were added into 1 g ± 0.1 g of rice flour followed by 60 min digestion and then cooled down to room temperature. The digested sample was titrated by the Automatic Kjeldahl nitrogen analyzer (BUCHIK‐436).

The AC was measured using the method of Zeng et al. (2019). First, 100 mg rice flour was gelatinized in 9.0 ml of 1 M NaOH in a boiling water bath for 10 min, to disperse the starch. Then, the solution was cooled down to room temperature and was diluted to the 100 ml volumetric flask. Finally, 3 ml reagent (1.0 ml CH_3_CH_2_OH and 2.0 ml I_2_) was added to 5 ml of the above solution, which was then measured at 720 nm of absorbance.

#### 2.2.3 Texture analyzers (TA) and RVA

The texture data of the rice were measured using a TAXT Plus texture analyzer (Stable Micro Systems) with a 36R probe. Texture analyzer for measuring textural properties were pretest speed 1 mm/s; post‐test speed 1 mm/s; test speed 0.5 mm/s; compression ratio 75%; trigger force 10.0 g; 8 parallels for each set of data (Li et al., 2018).

Pasting properties of the *japonica* rice samples were determined using a Rapid Visco Analyzer (RVA 4500, Perten Instruments). The rice flour (3.00 g, dry basis) was mixed with 25 ml of distilled water in an aluminum canister and tested following the procedures: First, heated to 50°C for 1 min, then heated to 95°C at 12°C/min in 3.75 min, maintained at 95°C for 2.5 min, cooled to 50°C at 12°C/min in 3.75 min, and finally maintained at 50°C for 1 min. The parameters including peak viscosity, trough viscosity, final viscosity, and pasting temperature were obtained.

### 2.3 Statistical analysis

The difference for the data (including HRR, CD, AC, Protein, TA and RVA) between the rice cultivars was analyzed systematically to calculate the least significant difference (LSD) values at the .05 probability level using IBM SPSS Statistics 22.0 (IBM Corp.). The analysis of variance (ANOVA) of the appearance (CD, HRR), composition (AC and Protein), and processing (TA and RVA) was tested the mean significance at the .05 probability levels, according to the least significant difference (LSD). Origin 9.0 (Origin Lab 2010) was used to draw the graph related to the article.

## 3 RESULTS AND DISCUSSION

Jiangsu province is becoming one of the largest producers of *japonica* rice in recent years. It is important to investigate the effect of different regional environments on the quality of different cultivars *japonica* rice to improve the economic value of rice. In order to investigate the cultivars difference and region effect, all samples were divided into 4 regions and 11 cultivars and summarized in Tables 2 and 3.

**TABLE 2:** Determination of rice quality in different regions

Parameter	RuDong	HaiAn	XingHua	LiShui
Appearance				
Chalkiness degree (%)				
Mean ± *SD*	15.34 ± 11.05a	15.34 ± 11.48a	11.68 ± 6.77a	6.81 ± 3.55b
Range	1.6–36.1	1.7–42.7	5.9–25	1.5–13.4
Head rice yield (%)				
Mean ± *SD*	94.00 ± 2.87a	89.10 ± 4.81b	94.52 ± 3.45a	94.79 ± 2.14a
Range	88.1–98.1	80.5–97.1	84.7–97.8	90.4–98.3
Taste value				
Mean ± *SD*	77.21 ± 2.06a	75.45 ± 2.44b	75.21 ± 2.87b	78.11 ± 3.56a
Range	73–81	68–79	70–79	70–83
Composition				
Protein (%)				
Mean ± *SD*	8.31 ± 0.51b	9.49 ± 0.88a	8.49 ± 0.67b	7.48 ± 0.96c
Range	7.7–9.5	7.7–11	7.2–9.6	6.1–9.6
AC (%)				
Mean ± *SD*	10.95 ± 2.35bc	9.96 ± 1.02c	11.63 ± 2.76ab	12.78 ± 3.17a
Range	9–17.82	8.06–12.12	9.37–17.46	9.97–17.69
Textural properties				
Hardness (g)				
Mean ± *SD*	1,801.99 ± 397.61b	1,947.27 ± 459.52b	2,318.58 ± 442.85a	1,817.53 ± 429.08b
Range	1,310.01–2,784.57	1,002.55–2,796.01	1,605.82–3,153.44	1,015.31–2,806.19
Resilience				
Mean ± *SD*	0.56 ± 0.09b	0.68 ± 0.07a	0.69 ± 0.07a	0.68 ± 0.11a
Range	0.36–0.76	0.53–0.83	0.55–0.82	0.51–0.92
Cohesiveness				
Mean ± *SD*	0.37 ± 0.06ab	0.36 ± 0.01b	0.38 ± 0.01a	0.38 ± 0.02a
Range	0.24–0.49	0.34–0.39	0.36–0.41	0.34–0.43
Gumminess (g)				
Mean ± *SD*	684.49 ± 186.06b	724.68 ± 183.22b	938.68 ± 168.42a	693.06 ± 183.12b
Range	399.94–1,104.99	381.89–1,182.46	594.03–1,267.09	371.61–1,181.62
Chewiness (g)				
Mean ± *SD*	478.62 ± 307.58b	518.16 ± 154.78b	638.79 ± 192.96a	488.81 ± 139.78b
Range	196.23–1,315.86	208.6–794.54	303.12–965.8	283.19–793.19
RVA				
Peak viscosity(cP)				
Mean ± *SD*	4,062.72 ± 461.64a	3,694.24 ± 438.79b	3,651.13 ± 499.73b	3,850.31 ± 687.77ab
Range	3,265–5,021	2,972–4,795	2,927–4,287	2,827–5,171
Trough viscosity (cP)				
Mean ± *SD*	1,957.52 ± 271.31b	1,869.91 ± 221.24b	1,993.17 ± 251.35b	2,139.24 ± 269.81a
Range	1,324–2,613	1,418–2,278	1,531–2,608	1,686–2,761
Final viscosity (cP)				
Mean ± *SD*	2,761.61 ± 377.21b	2,669.94 ± 285.84b	2,858.75 ± 408.24b	3,142.98 ± 469.35a
Range	2,128–3,626	1,578–3,296	2,225–3,850	2,423–4,225
Pasting temperature (°C)				
Mean ± *SD*	72.28 ± 2.41b	74.05 ± 3.18b	74.52 ± 4.28ab	76.40 ± 6.03a
Range	69.85–80.35	69.15–84.3	70.65–83.5	65.7–88.3
Environment				
Accumulated temperature (°C)	4,110.1	4,306	4,242.4	4,306.3
Accumulated Rainfall(mm)	541.5	457	416.1	380
Average Relative Humidity	81.7	73.5	78.3	76.7

Note

The different lowercase letters followed by a value indicates significant differences at the 0.05 probability level.

**TABLE 3:** Determination of appearance and composition of different cultivars

Variety	Chalkiness degree (%)	Head rice yield (%)	Taste value	Protein (%)	AC (%)
NJ9108 (RD)	16.05 ± 10.14bc	94.26 ± 2.52a	77.00 ± 1.87bc	8.38 ± 0.38b	10.20 ± 0.64 cd
CNG10 (RD)	6.77 ± 5.17de	93.63 ± 3.71ab	76.83 ± 2.56bcd	8.55 ± 0.91b	13.68 ± 4.48b
NG8 (RD)	2.10 ± 0.46e	90.27 ± 0.42bc	74.67 ± 1.53cde	9.37 ± 0.23a	10.22 ± 0.15 cd
XF1 (RD)	28.31 ± 3.12a	92.03 ± 2.39ab	78.83 ± 1.94ab	8.01 ± 0.20b	10.83 ± 1.65c
NJ9108 (HA)	17.23 ± 11.78b	88.26 ± 4.78c	75.74 ± 2.55cde	9.47 ± 0.97a	10.28 ± 0.82 cd
NJ3908 (HA)	6.86 ± 4.03de	92.85 ± 2.87ab	74.17 ± 1.33d	9.53 ± 0.10a	8.53 ± 0.35d
NJ9108 (XH)	12.42 ± 6.93bcd	94.89 ± 3.52a	75.90 ± 2.32cde	8.37 ± 0.63b	10.81 ± 1.74c
HD5 (XH)	6.47 ± 0.51de	91.93 ± 0.99ab	70.33 ± 0.58e	9.33 ± 0.06a	17.40 ± 0.05a
NJ46 (LS)	8.09 ± 2.79cde	94.73 ± 2.27a	80.48 ± 2.22a	6.91 ± 0.73c	11.33 ± 2.01c
PJ (LS)	6.38 ± 3.83de	95.88 ± 1.32a	74.25 ± 2.01de	8.43 ± 0.61b	17.27 ± 0.25a
NJ5055 (LS)	1.93 ± 0.28e	92.92 ± 1.56ab	75.17 ± 0.75cde	8.20 ± 0.09b	10.29 ± 0.13 cd

Note

The different lowercase letters followed by a value indicates significant differences at the .05 probability level.

### 3.1 Chalkiness degree and head rice rate difference analysis

Nowadays, consumers preferred high‐quality rice (Su et al., 2019). Chalkiness and head rice rate directly affect the economic quality of rice. Low chalkiness degree and high head rice rate are beneficial to improve the rice appearance quality. High‐quality *japonica* rice had good quality traits, and the rate of head rice would be a little changed (Zeng et al., 2019).

The results of CD and HRR obtained from four regions revealed a significant difference. The average chalkiness degree of four regions ranged from 6.81% to 15.34%. The difference between the maximum and minimum chalkiness degree ranged from 1.5% for LiShui region to 42.7% for Hai’An region (Table 2). For the differently degrees of chalkiness degree showed during the study results, 11 cultivars could be divided into three degrees (≥10%, 4%–10%, and ≤4%). The samples from RuDong region (NJ9108, CNG10, NG8, and XF1) had difference among cultivars and were divided into three degrees for above classification criteria (Table 3). The chalkiness degree of XF1, NJ9108 showed significantly higher than other cultivars, and the chalkiness degree of NJ9108 was similar in three different regions of Jiangsu (*p* <.05). Comparing with XF1 and NJ9108, NG8 and NJ5055 showed significant lower chalkiness degree (Table 3). The range of head rice rate in Hai 'an and XingHua regions was greater than 13%. The minimum head rice rate of cultivars from four regions was 80.5%. The head rice rate in RuDong and LiShui regions had less fluctuation than that Hai’An and XingHua regions, with changed range <10%. The head rice rate of all cultivars, except NJ9108 (HA), was higher than 90%.

Recent research had proved that genes, irrigation, carbon dioxide concentration, and environmental factors would affect *japonica* rice quality (Cheng et al., 2019; Liu et al., 2013). These cultivars with low chalkiness degree were adapted to the local planting environment, and these cultivars could be selected for further planting (Takai et al., 2019). In the present study, the range of tested varieties for chalkiness degree varied between cultivars and regions. In general, the *japonica* rice chalkiness degree range in LiShui region was comparatively lower than the other regions. It might be due to a lower rainfall and the cultivars difference. Furthermore, the large range of *japonica* rice chalkiness degree also showed the ability of varieties for heat resistance and ecological adaptability (Cheng et al., 2019). NJ9108 showed no significant difference chalkiness degree appearance trait among three regions, which indicated that the ability of high‐quality cultivars for ecological adaptability had same trend (Table 3). According to the data of Tables 2 and 3, the cultivars were easily to cause chalky difference than the environment. Therefore, it was more reasonable to evaluate the quality of *japonica* rice combined with its variety characteristics in different regions.

**FIGURE 2:**
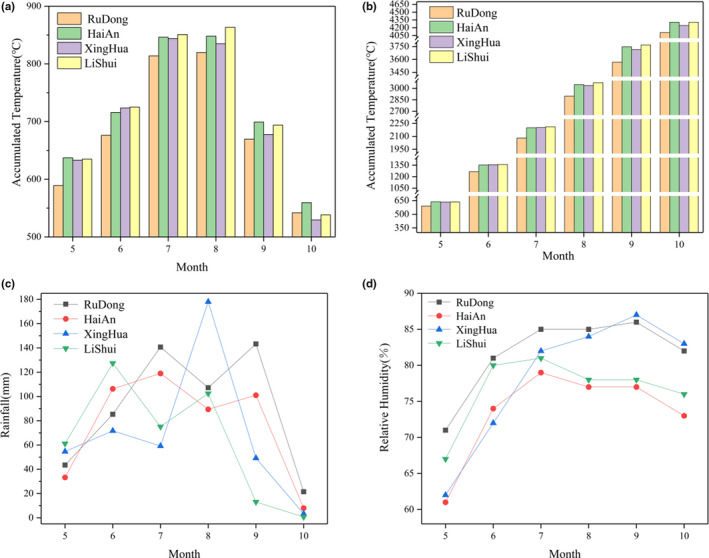
The environment statistics of four regions. The (a), (b), (c), and (d) correspond to effective accumulated monthly temperature, total accumulated temperature, rainfall, and relative humidity, respectively

The environment has an effect on the head rice rate of the same cultivar. During the growth stage, environmental condition is dynamic and has certain effects on the chalkiness and the rate of head rice (Lisle et al., 2000; Wang, et al.,^2015^). The relative humidity of Hai’An region was lower than RuDong and XingHua regions, and the accumulated temperature of Hai’An region was also higher than these two places (Figure 2b,d), Tang et al. (2018) mentioned that elevated temperature would destroy the original protein metabolic balance of the rice system and affect the appearance quality. This above phenomenon could explain the differences between protein content and chalkiness degree of NJ9108 (Table 3).

### 3.2 The taste value analysis

When assessing the edible quality of rice, personal preferences and other factors will affect assessment, so the rapid and nondestructive determination of the edible quality of rice by instruments is widely used (Kexin et al., 2014; Sun et al., 2015). The taste meter based on near‐infrared principle was used to determine the taste quality of rice with the characteristics of objectivity, convenience and nondestructive (Qingyun et al., 2007; Zhu et al., 2018). The JSWL taste meter equipped with a prediction model based on the characteristics of Chinese rice. Therefore, the taste values were valuable to evaluate the quality of *japonica* rice.

In Table 2, the taste values have no significant difference between RuDong and LiShui regions and the same for Hai’An and XingHua regions. The maximum taste values of cultivars from RuDong and LiShui regions were more than 80 (Table 2). These samples were also analyzed as based on cultivars. The whole cultivars except HD5 showed more than 74. For sample, the taste value of NJ46 reached 80.48 and was the highest among all cultivars. NJ46, HD5, and NJ3908 had a significant difference in the taste value (*p* <.05), but the taste values of other cultivars were close to each other (Table 3). The taste value of three NJ9108 samples showed inconspicuously difference between each other (*p* < .05), which indicated that NJ9108 has good adaptability to different environment.

### 3.3 Protein content and AC analysis

Starch and protein were the main components of whole grain rice and played an indispensable role in the texture and edible quality of rice grains (Zhou et al., 2002). The protein and AC of rice were closely related to the nutritional quality and cooking quality (Balindong et al., 2018; Bao, 2012; Bao et al., 2002).

The level of AC was determined by gen and affected by environment (Balindong et al., 2018; Tong et al., 2014). There were some differences in AC among different *japonica* rice cultivars and different environments in our study. The maximum AC in RuDong, XingHua, and LiShui regions reached 17% and higher than that in Hai’An region (Table 2). The AC of HD5, PJ, and CNG10 was more than 13%. The AC of other cultivars was around 10%. The AC of samples ranged from 8.53% to 17.40%, belonging to the low and medium AC. In addition to the high AC of HD5 and PJ, other cultivars were of low AC. In general, the AC was difference between cultivars in the same environment, but the influence of different environments did little to change in the same cultivar AC (Table 3). In addition, the AC of *japonica* rice is lower than *indica* rice and cultivars have a greater influence than environment (Kharabianmasouleh et al., 2012; Zeng et al., 2019).

The average of protein content in Hai’An region reached 9.49 and higher than other three regions, and the same trend of AC could be observed in LiShui region (Table 2). The protein content was closely to each other in RuDong and XingHua regions, and the result of AC was consistent in RuDong and XingHua regions. The difference between the maximum and minimum protein content ranged from 6.1% for LiShui region to 11% for Hai’An region (Table 2). Some cultivars also showed significantly difference in the protein content (*p* < .05). HD5, NJ9108 (HA), NJ3908, and NG8 were higher protein content than other cultivars and lowest for NJ46.

Environmental factors can lead to changes, during rice growth, in both composition and appearance (Liu et al., 2013; Tang et al., 2019). According to the environment data (Figure 2), the relative humidity in RuDong and XingHua from July to September was close. In Table 2, the protein content was closely to each other in RuDong and XingHua regions (*p* < .05). In addition, the cultivars of NG8 and HD5 were close in protein content (*p* < .05), and other cultivars in RuDong and XingHua regions also had same trend (Table 3). Considering some cultivars had good environment adaptability (Zeng et al., 2019), these results were explicable.

High temperature increased the protein content and chalkiness content of rice and decreased the head rate of whole rice (Liu et al., 2013). During the growth of *japonica* rice, high temperature would promote the development of protein in the endosperm of whole grain rice and increase the gap between starches, inducing the formation of chalkiness (Tang, Chen et al., 2018). NJ9108 (RD and XH) had significantly lower protein content and higher head rice yield than NJ9108 (HA) in Table 3. Effective accumulated monthly temperature and total accumulated temperature (Figure 2a,b) in Hai’An were higher than RuDong and XingHua, and the protein content and chalkiness degree had the same trend (Table 2), which were accorded with Liu et al. (2013) mentioned above. However, the sample from LiShui also has significantly lower protein content and higher head rice yield (Tables 2 and 3), but effective accumulated monthly temperature and total accumulated temperature were highest (Figure 2a,b). The reason for this abnormal result may be high‐quality *japonica* rice had good quality traits, good ability for heat resistance, and ecological adaptability (Cheng et al., 2019; Zeng et al., 2019). Thus, we could get the point that protein was influenced by both environment and cultivars, and higher adaptability *japonica* rice should be getting more attention in the further rice breeding, evaluation and market.

### 3.4 Textural properties analysis

Texture tests could quickly measure and reflect rice cooking and eating quality (Zohoun et al., 2018). Cooking rice was the process of intramolecular and intermolecular interactions of starch, protein, lipids, and nonstarch polysaccharides (Balindong et al., 2018).

Some textural properties of the cooked rice are also showed in Table 2. The hardness of XingHua region was higher than the other three regions, and XingHua region also had higher values for resilience, cohesiveness, gumminess, and chewiness: 0.69, 0.38, 938.68 (g), and 638.79 (g), respectively. Based on the analysis of hardness, gumminess, and chewiness, samples from RuDong, Hai’An, and LiShui regions had similar textural characteristic (Table 2). At the same time, based on the five textural indexes, NJ9108 (HA and XH), XF1, NJ3908, NJ46, and PJ had similar textural characteristic in a whole (Table 4). HD5 had a significantly higher hardness than others (*p* < .05). The same trends were observed in resilience and gumminess compared with other *japonica* rice, but no significantly difference was found in cohesiveness. The resilience and cohesiveness of XF1 and NJ9108 (HA) had been demonstrated whole nearly to each other. The resilience of NJ5055 was highest, but the gumminess of NJ5055 was lowest.

**TABLE 4:** Textural parameters of different rice cultivars by textural profile analysis

Variety	Hardness (g)	Resilience	Cohesiveness	Gumminess (g)	Chewiness (g)
NJ9108 (RD)	1,829.99 ± 312.11bcd	0.50 ± 0.07d	0.36 ± 0.05d	667.85 ± 163.89cde	352.30 ± 142.06e
CNG10 (RD)	1,685.94 ± 268.75 cd	0.60 ± 0.03bc	0.42 ± 0.07a	717.69 ± 99.52bcde	393.43 ± 85.34de
NG8 (RD)	1,441.31 ± 170.93d	0.57 ± 0.04 cd	0.41 ± 0.02ab	573.96 ± 57.25de	1,252.16 ± 78.65a
XF1 (RD)	2,014.97 ± 667.48bc	0.66 ± 0.10ab	0.36 ± 0.04d	756.44 ± 321.20bcd	556.01 ± 307.79 cd
NJ9108 (HA)	1,952.92 ± 450.43bc	0.68 ± 0.07ab	0.36 ± 0.01d	724.88 ± 185.76bcde	515.96 ± 166.93cde
NJ3908 (HA)	1,921.86 ± 543.54bc	0.71 ± 0.05a	0.37 ± 0.01 cd	723.75 ± 188.14bcde	528.02 ± 90.93cde
NJ9108 (XH)	2,206.53 ± 346.08b	0.68 ± 0.06ab	0.38 ± 0.02bcd	898.36 ± 136.94b	604.21 ± 179.88c
HD5 (XH)	3,102.95 ± 54.56a	0.77 ± 0.04a	0.40 ± 0.01abc	1,220.94 ± 45.95a	880.78 ± 64.78b
NJ46 (LS)	1,831.38 ± 454.93bcd	0.68 ± 0.11ab	0.37 ± 0.02 cd	679.57 ± 190.06cde	489.69 ± 137.55cde
PJ (LS)	1,991.13 ± 168.87bc	0.64 ± 0.12ab	0.39 ± 0.02abcd	808.32 ± 83.32bc	540.40 ± 143.81cde
NJ5055 (LS)	1,407.97 ± 428.93d	0.76 ± 0.02a	0.39 ± 0.01abcd	523.29 ± 156.57e	381.63 ± 88.69de

Note

The different lowercase letters followed by a value indicates significant differences at the .05 probability level.

In the rice cooking process, starch crystalline morphology changed to amorphous, rice with low AC was more prone to hydrolysis, and the contact area between whole grain rice and water increases, which leaded to soft and sticky rice. High amylose rice possessed opposite properties (Perdon et al., 1999). Liu et al. (2013) mentioned that the higher AC, the greater the hardness. Except LiShui region, RuDong, Hai’An, and XingHua regions had the same trend mentioned above (Table 2). This is due to the relative lower hardness of PJ (1,991.13 g) than that of HD5 (3,102.95 g). PJ and HD5 have the same AC, 17.27% and 17.40%. Texture characteristics of 9,108 collected from RuDong, Hai’An, and XingHua regions have slight fluctuations, indicating the stability of cultivars.

### 3.5 RVA analysis

**FIGURE 1:**
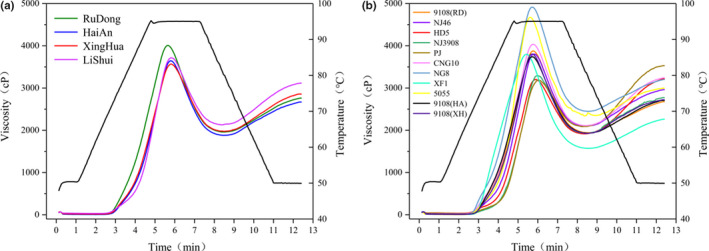
The RVA profiles of samples. The (a) and (b) correspond to four regions and different cultivars, respectively

Some information obtained from RVA analysis could be used to analyze rice quality (Taghinezhad et al., 2016; Wang et al., 2010). Previous studies have shown that RVA spectrogram analysis of rice mainly focuses on cultivars and RVA eigenvalues (Balindong et al., 2018; Cozzolino., 2016). Generally, the peak viscosity of RuDong region was higher than the other three regions, but the values of trough viscosity, final viscosity, and gelatinization temperature from LiShui region were higher (Table 2). In sum, starch content in rice from LiShui region was higher than rice from the other three regions, which was consistent with the RVA results. As can be seen in pasting curve, the highest viscosity was consistent between Hai’An, XingHua, and LiShui regions (Figure 1a). In addition, LiShui region had a different gelatinization curve with other three regions during the gelatinization progress when the temperature decreased.

Although the protein content and AC content have obviously difference, all samples from four regions had similar RVA curves (Figure 1a). The situation was the same between cultivars and corresponding RVA results, according to Table 5 and Figure 1b. Many factors could affect the RVA results, for example, protein, and starch secondary composition (Balindong et al., 2018).

**TABLE 5:** Rapid viscosity analyzer profile characteristics for different rice cultivars

Variety	Peak viscosity (cP)	Trough viscosity (cP)	Final viscosity (cP)	Pasting temperature (°C)
NJ9108 (RD)	3,994.11 ± 394.49b	1,954.72 ± 137.77c	2,659.38 ± 144.03c	73.17 ± 2.91 cd
CNG10 (RD)	4,039.17 ± 46.65b	2,107.50 ± 137.40bc	3,240.83 ± 339.37ab	71.07 ± 0.82 cd
NG8 (RD)	4,931.00 ± 97.15a	2,448.33 ± 158.86a	3,200.00 ± 173.52ab	70.15 ± 0.48d
XF1 (RD)	3,858.00 ± 555.49b	1,570.50 ± 162.11d	2,261.83 ± 80.54d	71.89 ± 0.47 cd
NJ9108 (HA)	3,782.44 ± 424.71bc	1,859.04 ± 228.56c	2,647.26 ± 305.67c	73.11 ± 1.46 cd
NJ3908 (HA)	3,297.33 ± 251.42 cd	1,918.83 ± 195.25c	2,772.00 ± 147.52c	78.27 ± 5.25b
NJ9108 (XH)	3,714.71 ± 503.08bcd	2,004.38 ± 266.52c	2,807.71 ± 411.78c	73.36 ± 3.11 cd
HD5 (XH)	3,206.00 ± 35.37d	1,914.67 ± 75.00c	3,216.00 ± 46.51ab	82.67 ± 0.83a
NJ46 (LS)	3,937.63 ± 580.67b	2,130.56 ± 311.24bc	3,004.07 ± 505.88bc	74.74 ± 4.85bc
PJ (LS)	3,199.25 ± 347.19d	2,082.08 ± 191.08bc	3,528.58 ± 225.08a	83.29 ± 4.39a
NJ5055 (LS)	4,759.50 ± 306.13a	2,292.67 ± 141.79ab	2,996.83 ± 98.07bc	71.66 ± 1.22 cd

Note

The different lowercase letters followed by a value indicates significant differences at the .05 probability level.

From through viscosity, the gelatinization curves of HD5, PJ, CNG10, and NG8 were similar and XF1 has the lowest (Figure 1b). The peak viscosity, trough viscosity, and final viscosity of NG8 were higher than that of most of other samples (*p* < .05) with 4,931.00, 2,448.33, and 3,200.00 cP. NG8 and NJ5055 had a higher viscosity than the other cultivars from 0 to 8 min in gelatinization progress before the trough viscosity formation. The gelatinization curves of 9,108 samples from three regions have similar gelatinization curves and exhibited slight difference in the peak viscosity with 3,994.11, 3,782.44, and 3,714.71 cP (Table 5 and Figure 1b).

### 3.6 Effect of region environment to rice quality

The interactions of cultivars and environments were the main factors to lead to the differences in texture characteristics (Zeng et al., 2019). Some research showed that paddy plant region is one of decisive factor in determining the quality of *japonica* rice (Yanjie et al., 2018), and rainfall is also an important factor on yield (Cornish et al., 2015). The difference of appearance and processing among four regions have showed the place of origin effected the quality of *japonica* rice (Table 2). LiShui region has the same trend of accumulated rainfall and relative humidity with RuDong and higher total accumulated temperature (Figure 2). Hai’An had lower relative humidity than other regions. Meanwhile, XingHua had the highest rainfall in August (Figure 2). AC was significantly negatively correlated with rainfall and relative humidity. RVA was also correlated with them negatively.

Akamatsu et al. (2020) revealed that the increase of temperature would cause the rise of gelatinization temperature. In our experiment, the increase of temperature has positively related to AC, resilience, and gelatinization temperature with 0.414, 0.498, and 0.281 (Table 6). The relationship was slight varied for different cultivars (Liu et al., 2017; Liu et al., 2015). In our experiment, AC was significant positive correlated with temperature, but the protein content had showed no significant correlated with temperature (Table 6). Rainfall and relative humidity both negatively related to AC with −0.304 and −0.407.

**TABLE 6:** Coefficient of correlations (*r*) between appearance, composition, textural, RVA, and environment

	Accumulated temperature	Rainfall	Relative Humidity
Appearance			
Chalkiness degree	−.219*	.35**	.056
Head rice yield	−.17*	−.136	.388**
Taste value	−.024	−.076	.126
Composition			
Protein	.008	.298**	−.282**
AC	.414**	−.304**	−.407**
Textural properties			
Hardness	.053	−.088	−.043
Resilience	.498**	−.461**	−.435**
Cohesiveness	−.064	−.065	.153
Gumminess	.033	−.108	.002
Chewiness	.038	−.07	−.023
RVA			
Peak viscosity	−.201*	.159	.206*
Trough viscosity	.104	−.257**	.056
Final viscosity	.173*	−.331**	.007
Pasting temperature	.281**	−.331**	−.175*

* Correlation is significant at 5% level.

** Correlation is significant at 1% level.

## 4 CONCLUSION

In this study, we collected 45 *japonica* rice samples from Jiangsu province and evaluated appearance and processing characteristics. The results indicated that the chalkiness degree had been presented significant differences, varied from 6.81% to 15.34% in Rudong, Hai'an, Xinghua, and Lishui and from 1.93% to 28.31% for different cultivars. We found that NJ9108 (HA) had a significantly lower head rice rate than other cultivars with 80.5%. The AC and protein content ranged from 8.53% to 17.4% and 6.1% to 11% according to different cultivars. Three 9,108 samples have similar gelatinization curves and texture characteristics, indicating the stability of cultivars. This experiment provides valuable data to establish *japonica* quality database for grain quality evaluation and promote the understanding the effect of cultivars, environment, and their interactions to appearance and processing characteristics of *japonica* rice.

## CONFLICTS OF INTEREST

Authors declare no conflict of interest.

## ETHICAL APPROVAL

Authors declare no studies involving animal and human subjects.

## Supporting information

Table S1‐S2
