# Supplementary material for: Grain quality evaluation of japonica rice effected by cultivars, environment, and their interactions based on appearance and processing characteristics
Source: Food Sci Nutr. 2021 Feb 24;9(4):2129–38. doi: 10.1002/fsn3.2181 (PMC8020948; doi:10.1002/fsn3.2181)
Supplement: Supplementary file 1 — Table S1‐S2 [file FSN3-9-2129-s001.docx]

*Supporting Information*

**Grain quality evaluation of** ***japonica* rice effected by** **cultivars, environment and their interactions based on** **appearance and processing characteristics**

Yujie Xia, Yuying Sun, Jian Yuan*, Changrui Xing*

College of Food Science and Engineering/Collaborative Innovation Center for Modern Grain Circulation and Safety/Key Laboratory of Grains and Oils Quality Control and Processing, Nanjing University of Finance and Economics, Nanjing 210023, People’s Republic of China

⁎ Corresponding authors. Tel: +86 025 86718509

yjian_nj@163.com

xingchangrui@nufe.edu.cn

**Table S1** Sample classification

| Region | Cultivars |
| --- | --- |
| Rudong | XF1, CNG10, NG8, NJ9108 |
| Hai’an | NJ9108, NJ3908 |
| Xinghua | NJ9108, HD5 |
| Lishui | NJ46, NJ5055, PJ |

**Table S2** Sample collection site

| Region | Cultivars | Longitude (E) | Latitude (N) |
| --- | --- | --- | --- |
| Rudong | XF1 | 121.03699  121.02704 | 32.30149  32.29885 |
|  | CNG10 | 121.03759  121.19361 | 32.30265  32.40665 |
|  | NG8 | 121.19522 | 32.40886 |
|  | 9108 | 121.04193  121.03631  121.19647  120.86050  121.18624  120.92414 | 32.29868  32.29689  32.40866  32.32797  32.32845  32.26587 |
| Hai’an | 9108 | 120.27086  120.35376  120.34307  120.38483  120.31965  120.31969  120.26219  120.26271  120.27045 | 32.57701  32.56767  32.39297  32.56686  32.50373  32.50329  32.63103  32.63078  32.58025 |
|  | 3908 | 120.34212  120.38494 | 32.39116  32.56688 |
| Xinghua | 9108 | 120.17464  120.17486  120.20344  119.93699  119.92949  119.92833  119.97254 | 32.93088  32.93139  32.92607  32.73811  32.82773  32.82764  32.87304 |
|  | HD5 | 119.92930 | 32.82726 |
| Lishui | NJ46 | 118.91680  119.00279  119.03377  119.03383  119.03063  119.03015  119.02128  119.05195  119.05362 | 31.55649  31.38650  31.43891  31.43762  31.43724  31.43765  31.44108  31.65222  31.65480 |
|  | NJ5055 | 118.92231  119.08615 | 31.55243  31.47962 |
|  | PJ | 118.91628  118.92031  118.92441  119.00779 | 31.55598  31.55355  31.55214  31.39408 |

The sample collection site was recorded by the Google map.
